# Blood preoperative neutrophil-to-lymphocyte ratio is correlated with TNM stage in patients with papillary thyroid cancer

**DOI:** 10.6061/clinics/2016(06)04

**Published:** 2016-06

**Authors:** Wenjie Gong, Shenjiu Yang, Xiumin Yang, Fang Guo

**Affiliations:** I Zhongshan Hospital Affiliated to Fudan University, Department of Hematology, Shanghai, China; II Zhangqiu People's Hospital, Jinan Sixth Municipal Hospital, Department of Pathology, Jinan, China; III Qian'an Maternal and Child Care Service Center, Department of Anesthesia, Tangshan, China

**Keywords:** Ratio of Neutrophils to lymphocytes, Papillary Thyroid Cancer, TNM Stage

## Abstract

**OBJECTIVE:**

To predict the American Joint Cancer Committee tumor-node-metastasis stage in patients with papillary thyroid carcinoma by evaluating the relationship between the preoperative neutrophil-to-lymphocyte ratio and the tumor-node-metastasis stage.

**METHODS:**

We retrospectively examined 161 patients with a diagnosis of papillary thyroid carcinoma. The Neutrophil-to-Lymphocyte Ratio was calculated according to the absolute neutrophil counts and absolute lymphocyte counts on routine blood tests obtained prior to surgery and patients with a Neutrophil-to-Lymphocyte Ratio of 2.0 or more were classified as the high NLR group, while those with a Neutrophil-to-Lymphocyte Ratio less than 2.0 were classified as the low Neutrophil-to-Lymphocyte Ratio group. Clinicopathological variables, which were stratified by the Neutrophil-to-Lymphocyte Ratio, were analyzed. A multivariate analysis was performed to determine factors that affect the Neutrophil-to-Lymphocyte Ratio. The association between the Neutrophil-to-Lymphocyte Ratio and the TNM stage in patients ≥45 years of age was analyzed using the Spearman rank correlation.

**RESULTS:**

Various blood indices, including hemoglobin, platelet and thyroid-stimulating hormone levels in the two groups showed no significant differences. Lymph node metastasis, multifocality and tumor size exhibited significant differences in the two groups (*p*=0.000, *p*=0.000 and *p*=0.035, respectively). Correlation analysis indicated that a higher preoperative Neutrophil-to-Lymphocyte Ratio was observed in patients with lymph node metastasis, larger tumor size and multifocality (r=0.341, *p*=0.000; r=0.271, *p*=0.000; and r=0.182, *p*=0.010, respectively). For patients ≥45 years of age, the number of patients with an advanced TNM stage in the high NLR group was higher than that in the low Neutrophil-to-Lymphocyte Ratio group (*p*=0.013). A linear regression analysis showed that the preoperative Neutrophil-to-Lymphocyte Ratio was positively correlated with the American Joint Cancer Committee tumor-node-metastasis stage (rho=0.403, *p*=0.000).

**CONCLUSION:**

The preoperative Neutrophil-to-Lymphocyte Ratio was closely related to the stage of papillary thyroid carcinoma. The increase in the preoperative Neutrophil-to-Lymphocyte Ratio contributed to the advanced tumor-node-metastasis stage of papillary thyroid carcinoma patients ≥45 years of age.

## INTRODUCTION

In recent years, many studies have found that the neutrophil-to-lymphocyte ratio (NLR) is related to the pathological characteristics of many tumors. The preoperative NLR is calculated according to the absolute neutrophil count (ANC) and the absolute lymphocyte count (ALC) of routine blood tests obtained prior to surgery. The systemic inflammatory response has been shown to promote tumor progression and metastases 1,2. It has been shown that inflammation is closely correlated with various stages of cancer development, including initiation, promotion, malignant conversion, invasion, and metastasis. The NLR is a biomarker that conveys information about inflammatory conditions. Thus, it is also useful for patients with solid cancer in various clinical settings. Ietomi found that the preoperative NLR can predict the progression of malignant tumors and proposed that the NLR is correlated with tumor prognosis 3. Many studies have shown that a high NLR is correlated with a high recurrence or fatality rate in lung cancer, gastric cancer, pancreatic cancer, colorectal cancer, hepatocellular carcinoma and breast cancer 4-9.

Thyroid cancer is the most common endocrine tumor. Its occurrence is associated with local and systemic inflammatory responses. Furthermore, thyroiditis can induce an immune reaction in the body 10,11. Thus, investigation of the relationship between the preoperative NLR in papillary thyroid cancer (PTC) patients and tumor characteristics is important for understanding tumor growth and the prognosis of thyroid cancer.

## METHODS

A total of 161 PTC patients, who were diagnosed according to pathology results after thyroid surgery between April 2014 and September 2015, were enrolled in this study.

The clinical parameters included age, gender, blood indices (ANC, ALC, hemoglobin and platelet levels), thyroid-stimulating hormone (TSH) levels, site of lymph node metastasis, tumor size and pathological features (e.g., multifocality), as well as the American Joint Cancer Committee (AJCC) tumor-node-metastasis (TNM) stage were collected. The tumor size was reported as the largest lesion dimension measured during a histopathological examination. The preoperative NLR was calculated by dividing the ANC by the ALC at the thyroid cancer diagnosis. We divided the cohort into two groups: NLR≥2 (high NLR group) and NLR<2 (low NLR group). The clinicopathological variables, which were stratified by the NLR were analyzed. Furthermore, a multivariate analysis was conducted to determine the factors that affect the NLR. Finally, the AJCC TNM stage of patients who were ≥45 years of age was further assessed using the NLR.

Continuous variables were compared using t-tests for two groups (when the variance was equal). The Mann Whitney test was used to compare continuous variables (when the variance was not equal). The Chi-square test was used to compare categorical variables. A multivariate analysis was performed to analyze the factors that affected the NLR. Spearman’s rank correlation was used to evaluate the association between the NLR and the AJCC TNM stage. All reported *p*-values were two-sided, except for the Pearson Correlation Coefficient (PCC) and were considered significant if *p* was less than 0.05. The calculations and construction of graphs were performed with SPSS 20.0 for Windows.

## RESULTS

The patient characteristics are shown in Table 1. The median age at presentation was 47 years (range 23-68 years). The median platelet (PLT) level was 235×10^9^/L (range 41-417×10^9^/L) and the median hemoglobin level was 131 g/L (range 79-170 g/L). The median ANC was 3.7×10^9^/L (range 1.8-8.3×10^9^/L), the median ALC was 1.9×10^9^/L (range 0.9-3.8×10^9^/L) and the median NLR was 2.0 (range 0.64-6.38).

Compared to the low NLR group, patients with PTC in the high NLR group were more likely to have a larger tumor (1.30±0.88 cm *vs*. 0.75±0.46 cm, *p*=0.000; Table 1 and Table 2), present with lymph node metastasis (52% *vs*. 25%, *p=*0.000; Table 1) and have multifocality (35% *vs*. 19%, *p=*0.000; Table 1) at diagnosis. No differences were observed between the two groups regarding age, sex, or PLT, hemoglobin and TSH levels.

In the multivariate analysis (Table 3), lymph node metastasis (r=0.341, *p*=0.000), tumor size (r=0.271, *p*=0.000) and multifocality (r=0.182, *p*=0.010) were strongly associated with the NLR. In addition, we determined that the standard regression coefficients (SRCs) of lymph node metastasis to the NRL in Model 1 and 2 were 0.341 (*p*=0.000) and 0.295 (*p*=0.000), respectively. The SRC of the tumor size to the NRL in Model 2 was 0.205 (*p*=0.007).

According to the AJCC TNM staging system, most patients <45 years were classified as stage I, while patients with distant metastases were classified as stage II. In contrast, patients >45 years of age with distant metastases were classified as stage IV 12. Thus, we limited the study to patients who were ≥45 years of age and evaluated the association between the AJCC TNM stage and the NLR. The number of patients with advanced TNM stage in the high NLR group was higher than that in the low NLR group (44% *vs*. 19%, *p*=0.013; Table 4). Moreover, Spearman’s rank correlation showed that the NLR was strongly correlated with the AJCC TNM stage, with a rho value of 0.403 (*p*=0.000; Table 5).

## DISCUSSION

Our study showed that the preoperative NLR was positively correlated with the size, lymph node metastasis and multifocality of malignant thyroid tumors. In addition, this study evaluated the preoperative NLR as a predictive factor for AJCC TNM stage in PTC patients >45 years of age. A higher preoperative NLR resulted in a larger tumor size and higher rates of lymph node metastasis and multifocality. All three of these aspects were correlated with the tumor burden. Thus, the preoperative NLR directly affected the TNM staging of thyroid cancer.

These results were also consistent with previous reports of other tumor types. The NLR has been previously assessed as an adverse prognostic factor for patients with cancers of the breast, lung, liver, colon and pancreas. In a meta-analysis that included over 40,000 patients with a variety of solid tumors, Templeton et al. showed that the NLR was associated with unfavorable overall survival 13.

The preoperative NLR might also reflect two interrelated processes, the systemic inflammatory response and the condition of the immune system.

An increase in the NLR reflects higher anti-inflammatory activity in patients with a high ANC and a low ALC. A correlation between a high NLR and elevated circulating pro-inflammatory cytokines, including interleukin-17 (IL-17) 14, interleukin-1 receptor α (IL-1R α), IL-6, IL-7, IL-8, IL-12 and monocyte chemotactic protein-1 15 was observed. These inflammatory cytokines may contribute to a tumor microenvironment that promotes tumor invasion 14,15,16. In addition, neutrophils can inhibit the secretion of tumor necrosis factor-α (TNF-α) and generate vascular endothelial growth factor (VEGF), which has been proposed to play a pivotal role in tumor development and angiogenesis 17,18. Tumor progression is induced by inflammatory cells, which can mediate biological mechanisms, such as angiogenesis, lymphangiogenesis and the secretion of survival and growth factors 19. Thus, an increased neutrophil level may exert adverse effects on the tumor-bearing host, resulting in an advanced TNM stage.

However, changes in the NLR can result in an immune system imbalance. The lymphocyte, the main component of tumor immunity, can induce natural killer lymphocytes and stimulate giant cells in tumors to release cytokines, including interferons and TNF-α, resulting in tumor shrinkage. The decrease in lymphocyte number might reflect reduced specific antitumor immune activity 20,21. Furthermore, tumor-associated macrophages have been associated with a poor prognosis. M2 macrophages promote cancer cell growth, as well as suppression of the antitumor immune response 22.

The NLR may contribute to a tumor growth imbalance and have a potentially important function in tumor development and the patient prognosis. The tumor likely exhibits uncontrolled growth, rapidly proliferates and may even metastasize into a more suitable microenvironment during the imbalance.

This study showed that the NLR was positively correlated with the AJCC TNM stage, which reflects the degree of malignancy of the tumor. An NLR≥2 was present in 52% of our patients and was associated with increased tumor size and a high rate of lymph node metastasis and multifocality. This ratio appears to be a useful and inexpensive predictive factor for PTC patients. Understandably, the present study has several limitations, such as its retrospective nature. Moreover, the optimal cut-off level of the NLR has not yet been standardized. We used a cut-off level of 2.0, although this level should be externally validated in independent cohorts. However, we propose that these results warrant further investigation in larger studies.

In conclusion, this study demonstrated a positive association between a high preoperative NLR and an advanced AJCC TNM stage in PTC patients >45 years of age. Taken together, these results indicate the importance of the NLR in assessing thyroid carcinoma and determining the corresponding treatment choice. This study provides a basis for enhancement of the lymphocyte value to decrease the NLR and control tumor growth through immune reactions.

## AUTHOR CONTRIBUTIONS

Gong W was responsible for analysis of the results, writing the manuscript and bibliographic research. Yang S was responsible for project supervision and data collection. Yang X was responsible for analysis of the results. Guo F was responsible for the bibliographic research and manuscript review.

## Figures and Tables

**Table 1:** **-** Patient characteristics according to the preoperative neutrophil-to-lymphocyte ratio..

	Total	NLR≥2	NLR<2	*p*-value
**Total**	161 (100%)	84 (52%)	77 (48%)	-
**Age**				
≥45 years	91 (57%)	48 (57%)	43 (56%)	0.708
<45 years	70 (43%)	36 (43%)	34 (44%)	
**Sex**				
Female	119 (74%)	62 (74%)	57 (74% )	0.975
Male	42 (26%)	22 (26%)	20 (26%)	
**PLT (10^9^/L)**	230.91±62.62	231.98±59.36	241.39±60.84	0.326
**Hb (g/L)**	139.10±31.00	134.39±12.60	132.87±16.17	0.505
**TSH level (uIU/ml)**	1.95±1.30	1.97±1.14	1.91±1.46	0.779
**Tumor size (cm)**	1.04±0.76	1.30±0.88	0.75±0.46	0.000
**Lymph node metastasis**				
Yes	63 (39%)	44 (52%)	19 (25%)	0.000
No	98 (61%)	40 (48%)	58 (75%)	
**Multifocality**				
Yes	44 (27%)	29 (35%)	15 (19%)	0.035
No	117 (73%)	55 (65%)	62 (81%)	

NLR: neutrophil-to-lymphocyte ratio; TSH: thyroid-stimulating hormone; TG: Thyroglobulin.

**Table 2:** Association between tumor size and the preoperative neutrophil-to-lymphocyte ratio.

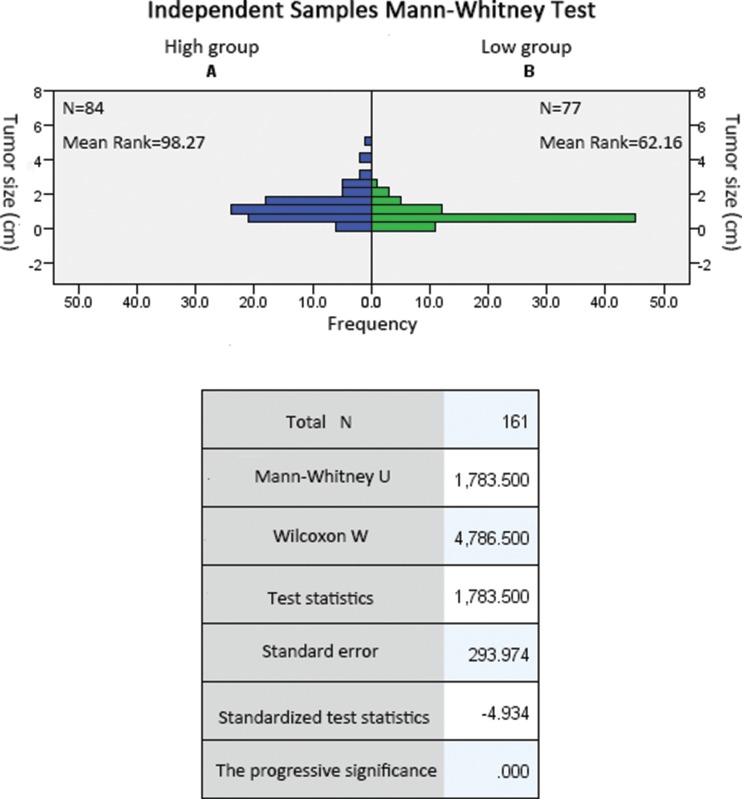

**Table 3:** Multivariate analysis of the preoperative neutrophilto-lymphocyte ratio.

	PCC/r	SRC (95% CI)	*p*-value (one-sided)
**Lymph node metastasis**	0.341		0.000
**Tumor size**	0.271		0.000
**Multifocality**	0.182		0.010
**Model 1**			
Lymph node metastasis		0.341 (0.323-0.814)	0.000
**Model 2**			
Lymph node metastasis		0.295 (0.245-0.739)	0.000
Tumor size		0.205 (0.061-0.379)	0.007

PCC: Pearson correlation coefficient; SRC: Standard regression coefficient.

**Table 4:** American Joint Cancer Committee TNM stage and neutrophil-to-lymphocyte ratio in patients ≥45 years of age.

	Total (%)	NLR≥2 (%)	NLR<2 (%)	*p*-value
**AJCC TNM Stage (age≥45 years)**				
Early (I or II)	61 (67)	27 (54)	35 (81)	0.013
Advanced (III or IV)	30 (33)	21 (44)	8 (19)	

**Table 5:** A linear regression analysis of the American Joint Cancer Committee TNM stage and the NLR in patients ≥45 years of age.

	American Joint Cancer Committee TNM stage	*p*-value
Spearman’s rho	0.403	0.000
